# Absence of signal peptide peptidase in peripheral sensory neurons affects latency-reactivation in HSV-1 ocularly infected mice

**DOI:** 10.1371/journal.ppat.1010281

**Published:** 2022-01-31

**Authors:** Shaohui Wang, Ujjaldeep Jaggi, Kati Tormanen, Satoshi Hirose, Homayon Ghiasi

**Affiliations:** Center for Neurobiology & Vaccine Development, Ophthalmology Research, Department of Surgery, Cedars-Sinai Medical Center, Los Angeles, California, United States of America; State University of New York Upstate Medical University, UNITED STATES

## Abstract

We previously reported that HSV-1 infectivity *in vitro* and *in vivo* requires HSV glycoprotein K (gK) binding to the ER signal peptide peptidase (SPP). Anterograde-retrograde transport via peripheral nerves between the site of infection (i.e., eye) and the site of latency (neurons) is a critical process to establish latency and subsequent viral reactivation. Given the essential role of neurons in HSV-1 latency-reactivation, we generated mice lacking SPP specifically in peripheral sensory neurons by crossing Advillin-Cre mice with SPP^fl/fl^ mice. Expression of SPP mRNA and protein were significantly lower in neurons of Avil-SPP^-/-^ mice than in control mice despite similar levels of HSV-1 replication in the eyes of Avil-SPP^-/-^ mice and control mice. Viral transcript levels in isolated neurons of infected mice on days 2 and 5 post infection were lower than in control mice. Significantly less LAT, gB, and PD-1 expression was seen during latency in isolated neurons and total trigeminal ganglia (TG) of Avil-SPP^-/-^ mice than in control mice. Finally, reduced latency and reduced T cell exhaustion in infected Avil-SPP^-/-^ mice correlated with slower and no reactivation. Overall, our results suggest that blocking SPP expression in peripheral sensory neurons does not affect primary virus replication or eye disease but does reduce latency-reactivation. Thus, blocking of gK binding to SPP may be a useful tool to reduce latency-reactivation.

## Author summary

HSV-1 gK and the ER protein SPP are both essential and highly conserved proteins. Their interaction is important for virus infectivity *in vitro* and *in vivo*. To evaluate the importance of gK binding to SPP in the peripheral nervous system, we generated SPP conditional knockout mice in peripheral nervous system using Advillin-Cre mice. The absence of SPP in peripheral nervous system significantly reduced latency-reactivation as well as T cell exhaustion.

## Introduction

HSV-1 encodes at least 80 genes [1], one of which, glycoprotein K (gK) [1–3], encodes a highly hydrophobic protein with a cleavable 30-aa N-terminal signal sequence, two glycosylation sites, and high protein homology with HSV-2 [1,4–7]. HSV-1 infections are among the most frequent serious viral eye infections in the U.S. [8]. We previously demonstrated the importance of gK in HSV-induced corneal scarring (CS) and facial dermatitis [9–15]. Furthermore, in both mice and humans, gK elicited CD8^+^IFN-γ^+^ responses [16,17]. We also found that CD8^+^ T cell depletion in gK immunized mice reduced gK-induced CS in ocularly infected mice [18]. In addition, mutation within the 8mer region of the gK N- terminus blocked cell surface expression of gK and reduced CS in ocularly infected mice [19]. Binding of gK to SPP is known to be essential for HSV-1 infectivity both *in vitro* and *in vivo* [20,21]. Collectively, these data strongly support the concept that gK plays a major role in viral immunopathogenesis.

Previously we found that gK binds to signal peptide peptidase (SPP) [21], leading to viral immunopathogenesis [20–22]. SPP (aka minor histocompatibility antigen H13) is an endoplasmic reticulum (ER-resident) protein that is highly conserved between vertebrates and non-vertebrates [23–29]. Overall, SPP functions in both immune surveillance and ER quality control [30–33]. Since SPP is an essential gene and SPP knockout mice are embryonically lethal [34], we previously depleted SPP in mice using a tamoxifen-inducible Cre recombinase [22]. Despite the incomplete depletion of SPP by tamoxifen, treated mice had significantly less HSV-1 replication in their eyes and also had reduced latency. Our results with tamoxifen-inducible Cre mice suggest that cleavage of gK N-terminal leader sequences is mediated by SPP after gK arrives in the ER. ER-directing parts of N-terminal leader peptides localize to the ER lumen where they can interact with MHC-I molecules to enhance CD8^+^ T cell responses and exacerbate CS [22].

In both animals and humans, HSV-1 travels via anterograde and retrograde transport between the site of infection and the ganglia of the infected hosts [35,36]. After initial infection, the virus establishes latency in peripheral sensory neurons of an infected host. At various times post infection (PI), the virus may reactivate, travel back to the original site of infection and cause recurrent disease [37–39]. Because of the importance of peripheral sensory neurons and established role of SPP interaction with gK during HSV- 1 latency-reactivation, we wanted to determine the effect of deleting SPP in peripheral sensory neurons. Our approach overcomes the problem of lack of tissue specificity with tamoxifen depletion of SPP as well as the embryonic lethality of SPP KO mice. Advillin is an actin regulatory/binding protein expressed at high levels in dorsal root ganglia and TG at embryonic stages and in adult animals [40]. Thus, we used and generated a Cre-driver mouse line that expresses Cre recombinase from the locus of the peripheral sensory neuron specific gene *Advillin*. *This conditional knockout mouse strain lacks SPP expression in* peripheral sensory neurons *and we* refer to these mice as Avil-SPP^-/-^, hereafter. *These mice are viable and appear normal except that they lack SPP gene expression in peripheral nerves but not any other tissues*.

In the present study, we investigated the impact of absence of SPP expression in peripheral sensory neurons as well as its impact on HSV-1 infectivity using Avil-SPP^-/-^ mice. We show that in the absence of SPP in peripheral sensory neurons: 1) virus replication was similar in the eyes of infected and control mice; 2) gB, gK and ICP0 transcripts in isolated neurons of infected Avil-SPP^-/-^ mice, but not in total TG, were expressed at lower levels than in control mice; 3) latency-reactivation in isolated neurons and total TG of latently infected Avil-SPP^-/-^ mice was significantly less than in control mice; and 4) CD8α T cell exhaustion in isolated neurons, but not in total TG RNA, of latently infected Avil-SPP^-/-^ mice was significantly less than in control mice. The absence of SPP in infected mouse neurons and its lack of binding to gK correlated with lower latency-reactivation and lower T cell exhaustion. Therefore, blocking gK-SPP binding may present an effective way to reduce latency -reactivation and thus, pathology associated with HSV-1 reactivation.

## Results

### Generation of Avil-SPP^-/-^ mice

**Fig 1:**
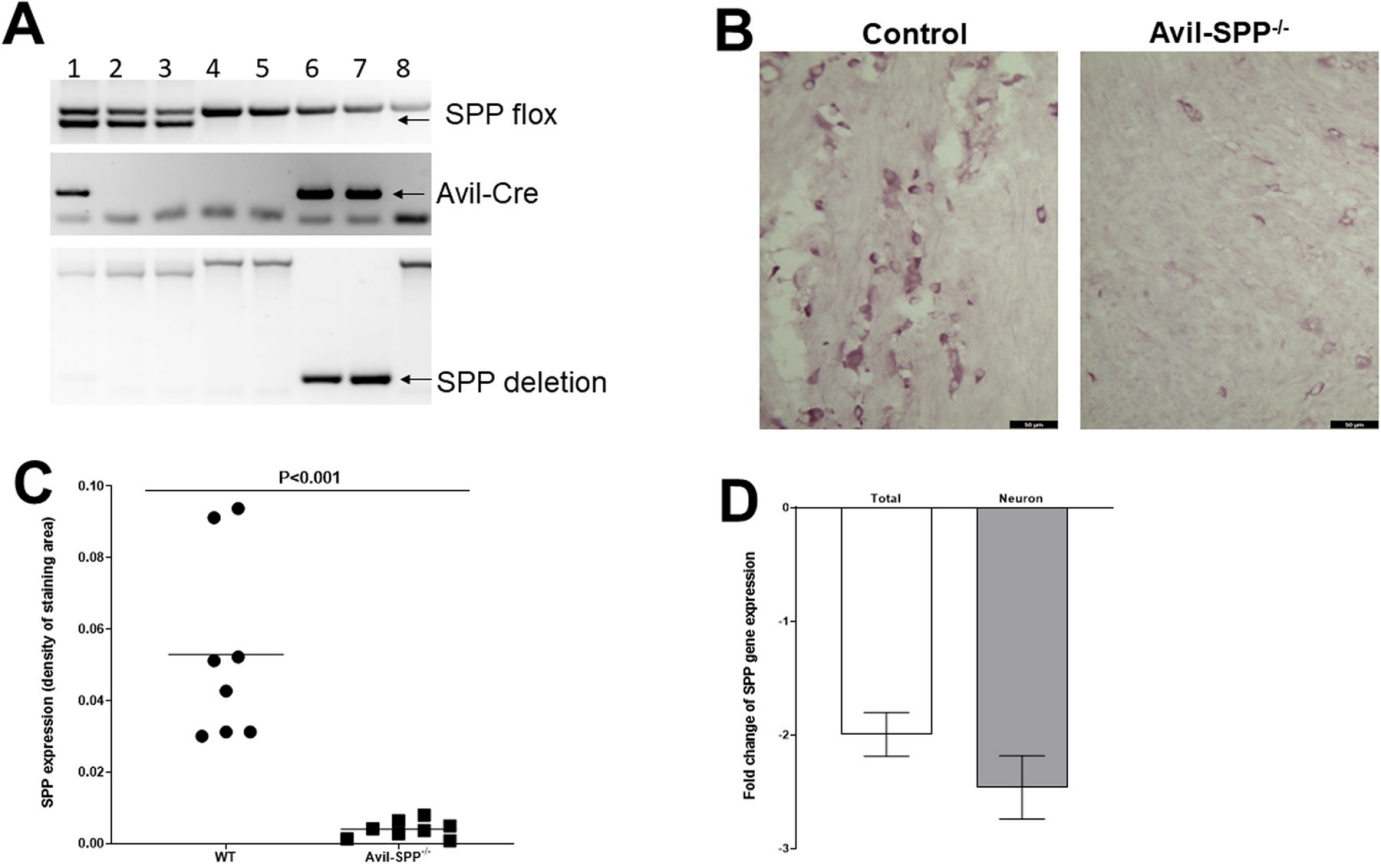
Development of sensory neuron specific SPP conditional knockout mice. (A) Confirmation of Avil-SPP^-/-^ moue genotype. Sensory neuron specific SPP knockout mice (Avil-SPP^-/-^) were generated by crossing SPP^flox/flox^ mice with Advillin-Cre^+^ mice to generate Avil-SPP^-/-^ mice, in which SPP was specifically knocked out in Advillin-expressing neuronal tissue. Mouse genotypes were confirmed by PCR as shown and mice 6 and 7 were confirmed to be Avil-SPP^-/-^ (SPP^flox/flox^Avil-Cre^+^); (B) Lack of SPP expression was confirmed in TG of Avil-SPP^-/-^ mice by IHC. TG of 2 month old Avil-SPP^-/-^ mice or control mice were harvested and frozen in OCT compound at -80°C for further processing. IHC was performed with anti-SPP antibody and developed by VectaShield VIP substrate kit. SPP positive cells (purple color) were more abundant in TG from Avil-SPP^-/-^ mice than from the control group; C) Quantitation of SPP expression density in TG of Avil-SPP^-/-^ mice. SPP expression from eight separate IHC staining as (B) above were quantitated by image analysis; and (D) Lack of SPP expression in TG of Avil-SPP^-/-^ mice confirmed by qRT-PCR. TG of 2-month-old Avil-SPP^-/-^ or control mice were harvested, total individual TG RNA or RNA from isolated neurons were extracted, and qRT-PCR was performed. Levels of RNA expression in total TG RNA or neuronal RNA were normalized to that of RNA from their respective control mice. Fold change represents the mean ± SEM from six replications (6 TG for total RNA isolation and 24 TG for RNA isolation from neurons).

We previously reported the important role of HSV-1 gK binding to SPP in HSV-1 infectivity *in vitro* and *in vivo* using SPP dominant-negative mutants, SPP shRNA, and chemical inhibitors of SPP [20,21]. Since neurons of the infected host are the final destination of HSV-1 infection, we wanted to determine the effects of SPP absence from peripheral sensory neurons on HSV-1 infectivity *in vivo*. To address this question, we generated mice lacking SPP in their sensory neurons by crossing SPP^fl/fl^ mice with mice expressing Cre recombinase under control of the Advillin promoter [41]. Breeding these mice produced mice lacking SPP specifically in peripheral sensory neurons, referred to as Avil-SPP^-/-^ mice (see Materials and Methods, Fig 1A). SPP protein expression was monitored by staining TG sections from Avil-SPP^-/-^ and control mice with anti-SPP antibody (Fig 1B). We observed less positive SPP staining in TG from Avil-SPP^-/-^ mice than in stained TG sections from control mice (Fig 1B, compare TG from control group with TG from Avil-SPP^-/-^ group). Density of SPP expression from the above IHC staining TG were quantified as described in Materials and Methods. There was a significant reduction in SPP expression level in TG of Avil-SPP^-/-^ mice compared to TG from wt mice (Fig 1C, p<0.001).

In human TG, 20,000 to 35,400 neurons, with an average of 27,400 ± 4,800, and about 100 times more non-neuronal cells have been reported [42]. Similarly in mouse TG, 22,500 to 25,583 neurons, with an average of 24,117 ± 1,542 neurons per TG have been reported [43,44]. Non-neuronal cells in the TG are mainly Schwann cells, satellite glial cells, fibroblasts forming collagen fibers, small blood vessels (mainly capillaries), and several types of immune cells [45,46]. Because Advillin is expressed at high levels in sensory neurons of the TG [40], we used qRT-PCR to compare SPP RNA expression in Avil-SPP^-/-^ and control mice. RNA levels in isolated neurons and in total TG from Avil-SPP^-/-^ mice were significantly less than in control mice (Fig 1D). Thus, Avil-SPP^-/-^ mice lack expression of SPP in their sensory neurons. These mice grow and breed normally, and no neuronal dysfunction was detected in these mice using esthesiometry and tonometry.

### Absence of neuronal SPP does not exacerbate eye disease or affect survival

**Fig 2:**
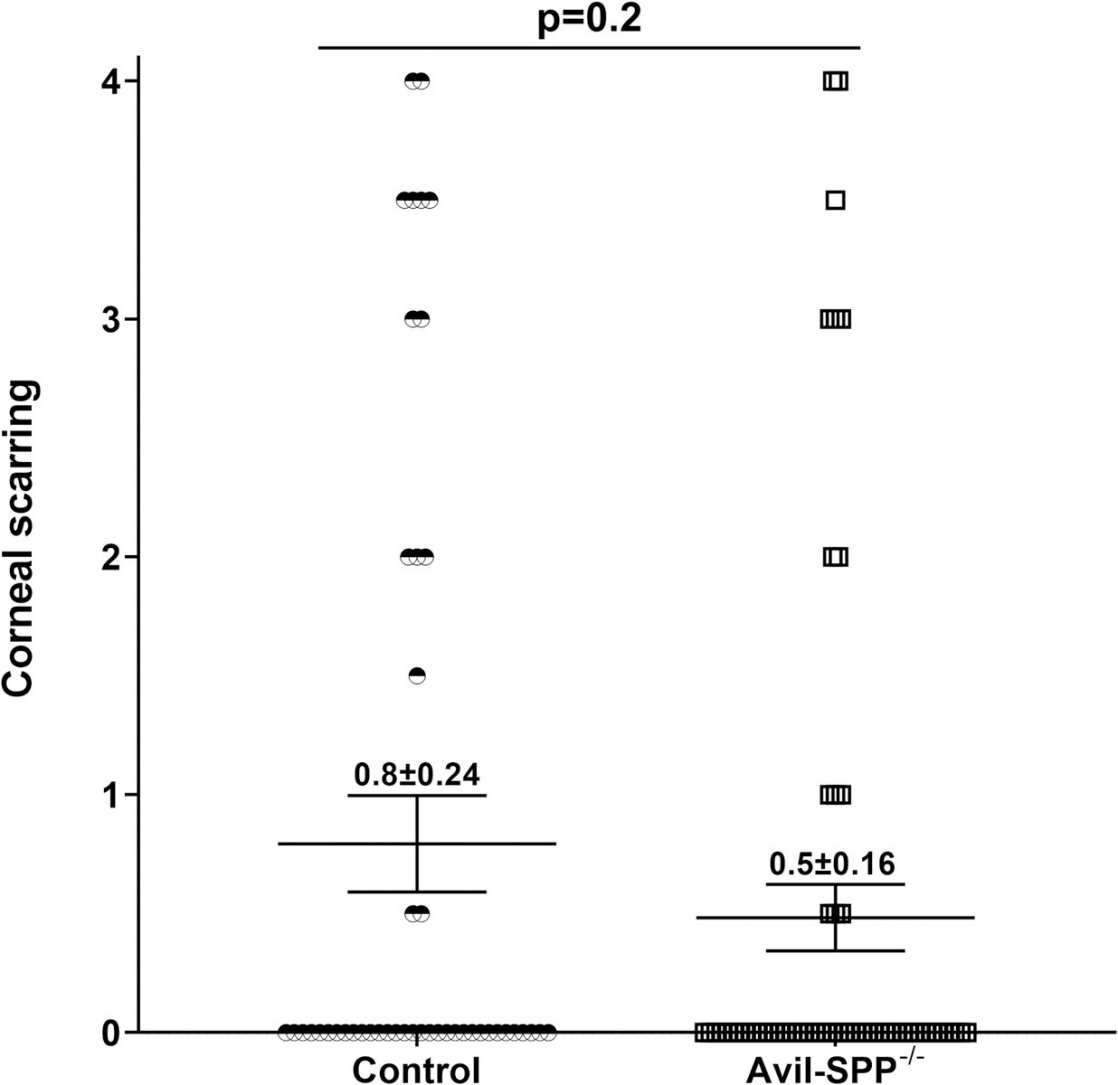
Effect of SPP deficiency on corneal scarring in infected mice. Avil-SPP^-/-^ and control mice were ocularly infected with 2 X 10^5^ PFU/eye of HSV-1 strain McKrae. Severity of corneal scarring was determined on day 28 PI. Each point represents the mean ± SEM from 46 eyes for control mice and 60 eyes for Avil-SPP^-/-^ mice.

A total of 30 Avil-SPP^-/-^ mice and 25 control mice (in four separate experiments) were ocularly infected with 2x10^5^ PFU/eye of HSV-1 strain McKrae as above. Survival of infected mice was recorded at day 28 PI. All (total n = 30) of the mice in the Avil-SPP^-/-^ group and 23 of 25 in the control group survived ocular infection. No significant differences in survival were detected between the Avil-SPP^-/-^ and control groups (p>0.05, Mann Whitney test). Eyes of the surviving mice were examined for corneal scarring (CS) at day 28 PI. There were no statistically significant differences in CS between the Avil-SPP^-/-^ and control groups (Fig 2; p = 0.2, Fisher’s t test). Thus, the absence of SPP in peripheral sensory neurons did not exacerbate CS or result in death of infected mice. These differences between this current study and our previous studies [20,21], is probably due to specific depletion of SPP in the neurons of Avil-SPP^-/-^ mice compared with blocking of SPP expression in all tissues.

### Virus replication in the eyes of ocularly infected Avil-SPP^-/-^ mice was not affected by the absence of SPP

**Fig 3:**
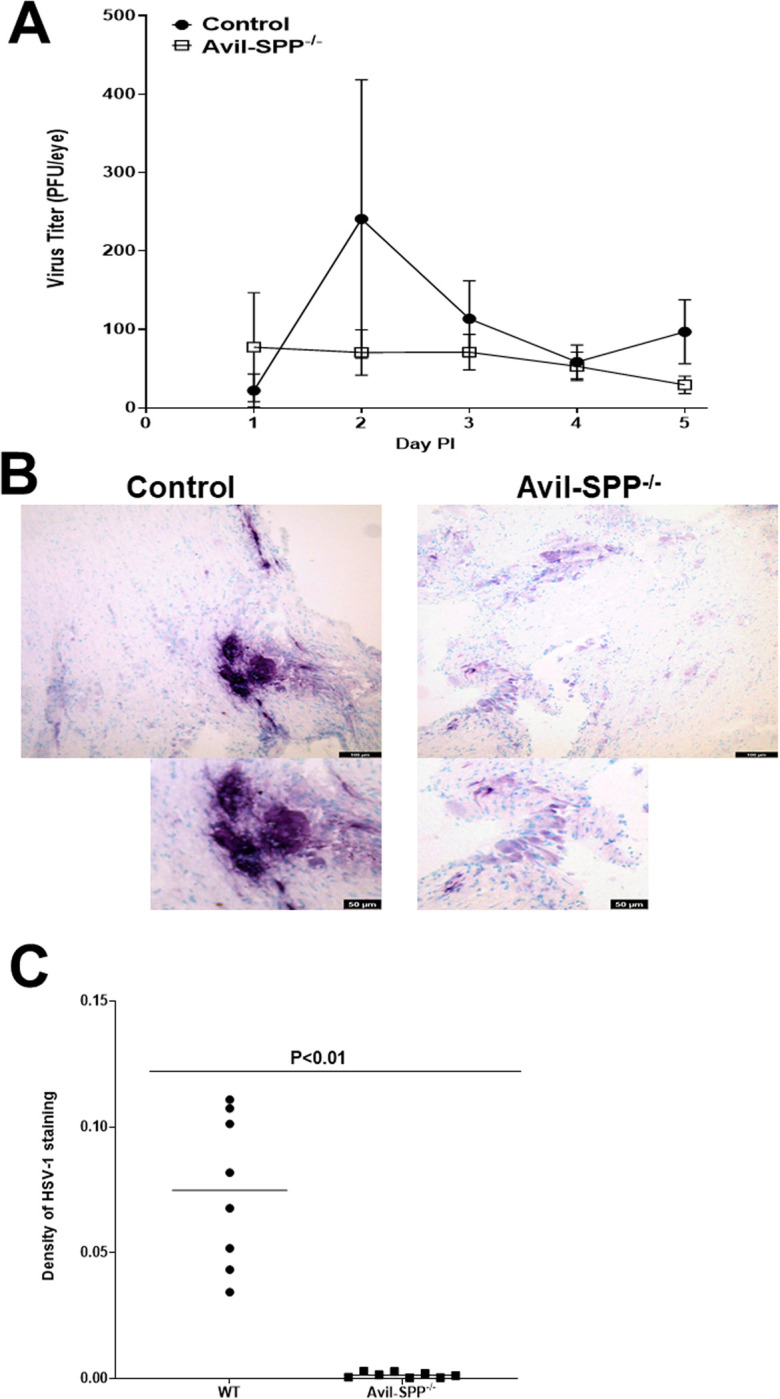
Virus titer in eyes of infected mice and SPP expression in TG of infected mice. (A) Virus titers in the eyes of Avil-SPP^-/-^ mice. Avil-SPP^-/-^ and control mice were ocularly infected with 2 X 10^5^ PFU/eye of HSV-1 strain McKrae. Tear films were collected on days 1 to 5, and virus titers were determined by standard plaque assay. Each point represents the mean titer of 26 eyes from two separate experiments; (B) HSV-1 protein expression in TG of infected mice. Avil-SPP^-/-^ and control mice were ocularly infected with 2 X 10^5^ PFU/eye of HSV-1 strain McKrae. TG were harvested on day 5 PI and frozen in OCT compound until processing. IHC was performed with anti-HSV-1 antibody and developed using a VectaShield VIP substrate kit. Staining of HSV-1 protein positive cells (purple color) was much stronger in the control group than in the Avil-SPP^-/-^ group; and C) Quantitation of HSV-1 expression density in TG of infected Avil-SPP^-/-^ mice. HSV-1 expression from eight separate IHC staining as (B) above were quantitated by image analysis.

To determine if the absence of SPP in peripheral sensory neurons alters virus replication in the eyes of infected mice, Avil-SPP^-/-^ and control mice were ocularly infected with HSV-1 strain McKrae. Tear films were collected from 26 eyes/group in two separate experiments on days 1, 2, 3, 4, and 5 PI and the presence of infectious virus was determined by plaque assays (Fig 3A). Virus titers in the eyes of the two infected mouse groups were similar on days 1 to 5 PI (Fig 3A, P>0.05 at all-time points). Thus, the absence of SPP in Avil-SPP^-/-^ mice was not associated with reduced virus replication in the eyes of infected mice. This result conflicts with our previous work showing that the global absence of SPP in SPP-inducible knockout mice was associated with reduced virus replication in the eyes of infected mice [22]. Furthermore, in contrast to our current study and in line with our previous study in SPP-inducible knockout mice, blocking SPP binding to gK using SPP dominant-negative mutants or shRNA against SPP significantly reduced HSV-1 replication *in vitro* [21]. We attribute this discrepancy between our current and previous studies to the fact that in our previous study tamoxifen treatment, SPP dominant-negative mutants, or shRNA against SPP, depleted and blocked SPP in all tissues, while in this study SPP was expressed in all tissues but was selectively blocked in peripheral sensory neurons.

### Absence of SPP expression in Avil-SPP^-/-^ mice reduces HSV-1 protein expression in TG of infected mice

Fig 1B showed no detectable SPP protein expression in TG of naive Avil-SPP^-/-^ mice compared with control mice. We next tested if the absence of SPP affected HSV-1 protein expression in TG of Avil-SPP^-/-^ mice compared with control mice. Avil-SPP^-/-^ and control mice were ocularly infected with HSV-1 strain McKrae and TG of infected mice were isolated on day 5 PI, sectioned and stained with anti-HSV-1 antibody. Immunostaining showed markedly fewer HSV-1^+^ cells in TG of Avil-SPP^-/-^ mice than in TG of control mice (Fig 3B). Density of HSV-1 expression from the above IHC staining were quantified as described in Materials and Methods. There was a significant reduction in HSV-1 expression level in Avil-SPP^-/-^ infected TG compared to infected TG from wt mice (Fig 3C, p<0.01). These results suggest that the absence of SPP expression significantly reduced HSV-1 protein expression in TG of Avil-SPP^-/-^ mice.

### Expression of gK, ICP0 and gB transcripts are altered in neuron of Avil-SPP^-/-^ mice during primary ocular infection

**Fig 4:**
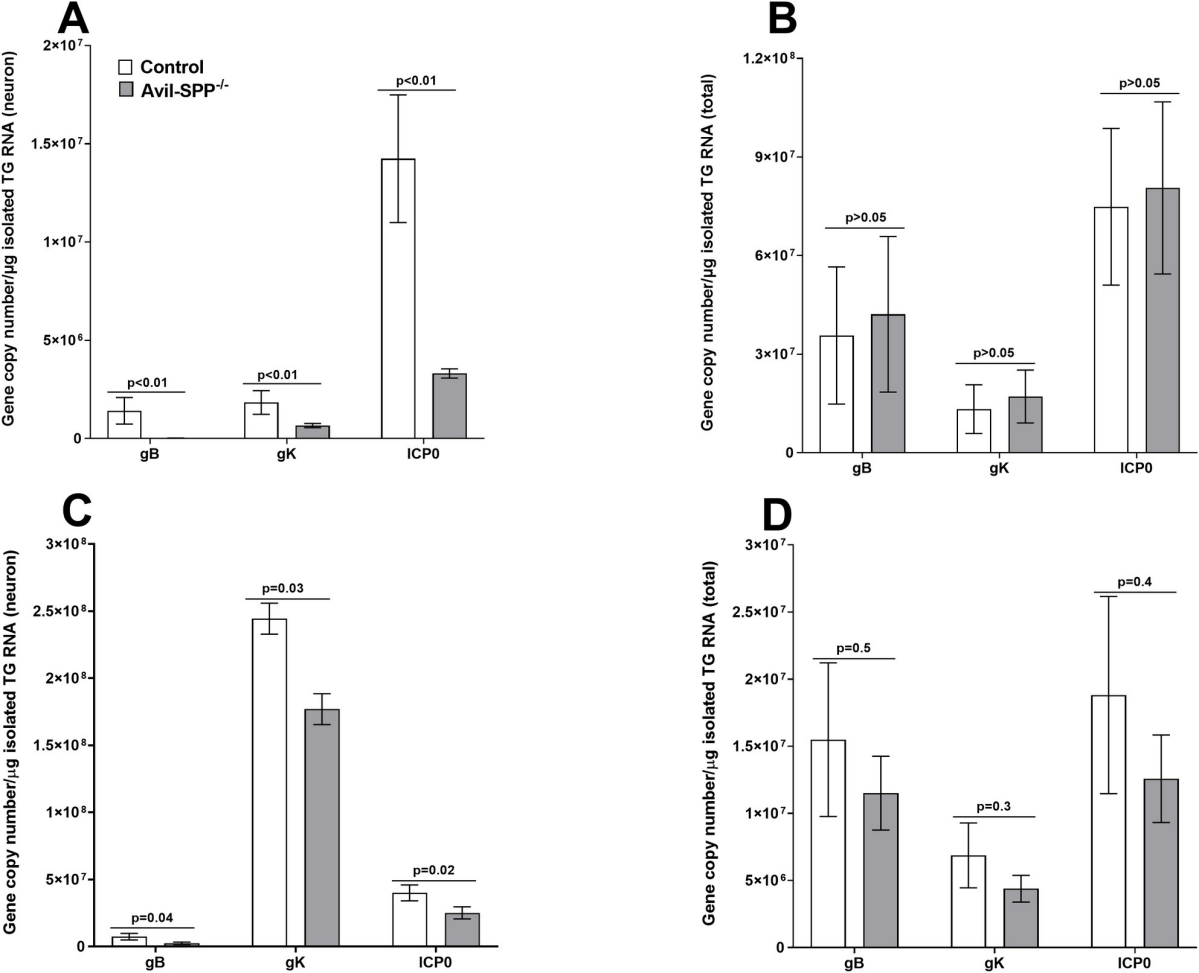
Expression of gB, gK, and ICP0 transcripts in TG of Avil-SPP^-/-^ infected mice. Avil-SPP^-/-^ and control mice were ocularly infected with 2 X 10^5^ PFU/eye of HSV-1 strain McKrae. TG were harvested on days 2 and 5 PI. RNA from individual TG or from the neuron fraction of four combined TG were isolated as described in Materials and Methods. gB, gK and ICP0 transcripts were measured by qRT-PCR and copy numbers of each transcript were calculated using standard curves generated using their respective plasmids. Neuronal RNA was isolated from four combined TG, while total TG RNA was isolated from individual TG. Each bar represents mean ± SEM from 12 mouse TG for neurons (A and C), and 12 TG (B and D) from six mice for total TG RNA.

**Fig 5:**
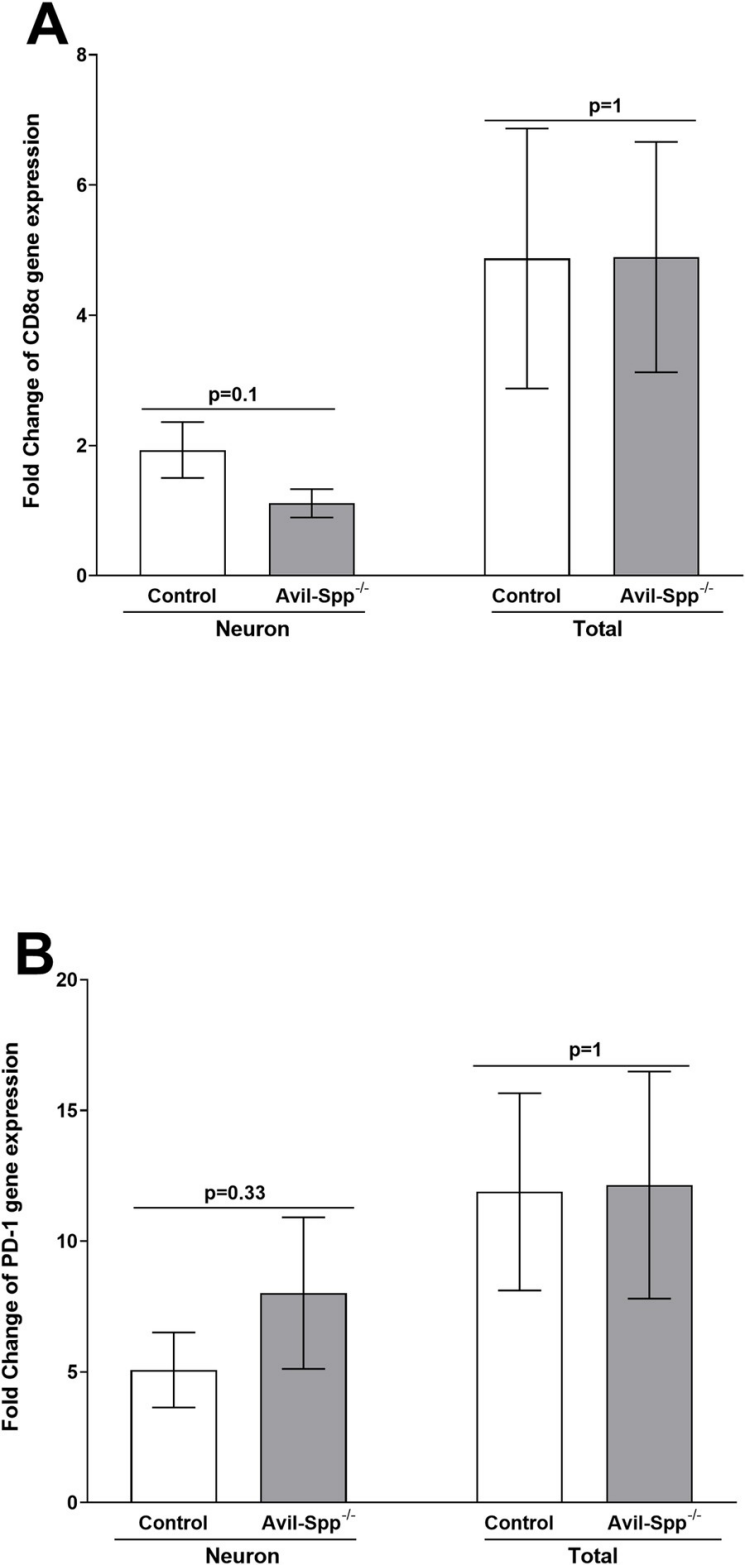
Expression of CD8α and PD-1 transcripts in TG of Avil-SPP^-/-^ infected mice during primary infection. Isolated RNA from total TG and neuronal fraction (see Fig 3) was used to measure CD8α and PD-1 transcript expression by qRT-PCR. The ratio of expression for each mRNA transcript was normalized to its expression in its respective uninfected group. GAPDH expression was used to normalize relative expression of each transcript. Neuronal RNA was isolated from four combined TG, while total TG RNA was isolated from individual TG. Each bar represents the mean ± SEM from 12 mouse TG for neurons and 12 TG from six mice for total TG RNA.

As expected, Fig 3A showed that the absence of SPP in peripheral sensory neurons did not affect virus replication in the eyes of infected mice. We next asked whether the lack of SPP in neurons affected expression of viral transcripts, especially gK, in TG of infected mice. Avil-SPP^-/-^ and control mice were infected as above with HSV-1 McKrae. On days 2 and 5 PI, TG from infected mice were collected and divided into two groups: in one group TG were fractionated into neuronal cells and their RNA was isolated, whereas in the second group, total RNA was isolated from the whole TG without fractionization. RNA isolated from neurons and total TG were analyzed by TaqMan RT-PCR to determine the copy numbers for gK, gB and ICP0 mRNAs. GAPDH mRNA in each sample was used as an internal control. The results showed lower levels of each transcript in TG of infected Avil-SPP^-/-^ mice than in control mice on both days 2 (Fig 4A) and 5 (Fig 4C). On both days 2 and 5, isolated neurons from TG of infected Avil-SPP^-/-^ mice, gB, gK, and ICP0 transcripts were significantly lower than in control mice (Fig 4, panels A and C, Fisher’s exact test). However, while levels of gB, gK, and ICP0 transcripts were significantly lower in Avil-SPP^-/-^ neurons than control mice on both days 2 and 5 post ocular infection, they did not significantly differ in total RNA isolated from TG on day 2 (Fig 4B, Fisher’s exact test) and day 5 (Fig 4D, Fisher’s exact test). The lack of significant differences between transcripts from control and Avil-SPP^-/-^ mice (Fig 4, panels B and D) is probably due to use of total RNA from a single TG rather than combining four TG (Fig 4, panels A and C), meaning that transcript copy number was measured per total RNA, leading to a lower concentration of viral RNA than that from purified neurons. Overall, more viral transcripts were detected in isolated neurons than in RNA isolated from total TG extract (Fig 4, compare panels A and C with B and D). Finally, the expression levels of gK transcripts for both groups were higher in comparison to gB or ICP0 transcripts isolated from neurons in contrast to ICP0 transcripts which were higher than expression levels for gB or gK transcripts in total RNA isolated from TG. Collectively, these results indicated that the absence of SPP in neurons of infected mice is associated with reduced viral transcripts in neuronal cells.

### Absence of SPP in Avil-SPP^-/-^ mice does not alter CD8α and PD-1 expression in TG of infected mice during primary infection

The above result suggested that the absence of SPP affects viral transcripts in TG of infected mice but does not affect virus replication in the eye of infected mice compared with control mice (Figs 3 and 4). Therefore, we asked whether lower viral transcript levels in Avil-SPP^-/-^ mice affects expression levels of CD8α and therefore PD-1 in TG of infected mice. RNA isolated from Avil-SPP^-/-^ and control mice (see Fig 4) was used to measure CD8α and PD-1 transcript levels in TG of infected mice by qRT-PCR. Results are presented as “fold” increase over baseline mRNA levels in TG of naive mice for each group. GAPDH mRNA in each sample was used as an internal control. CD8α transcripts in isolated neuronal or total TG RNA were similar in mice with and without SPP expression (Fig 5A, p>0.05, Fisher’s exact test). PD-1 transcripts levels were also similar in isolated neuronal or total TG RNA from Avil-SPP^-/-^ or control mice (Fig 5B, p>0.05, Fisher’s exact test). These results suggest that absence of neuronal SPP or lower expression of viral transcripts does not affect expression levels of CD8α and PD-1 in TG during acute viral infection.

### Latency is reduced in the TG of latently infected mice lacking SPP

**Fig 6:**
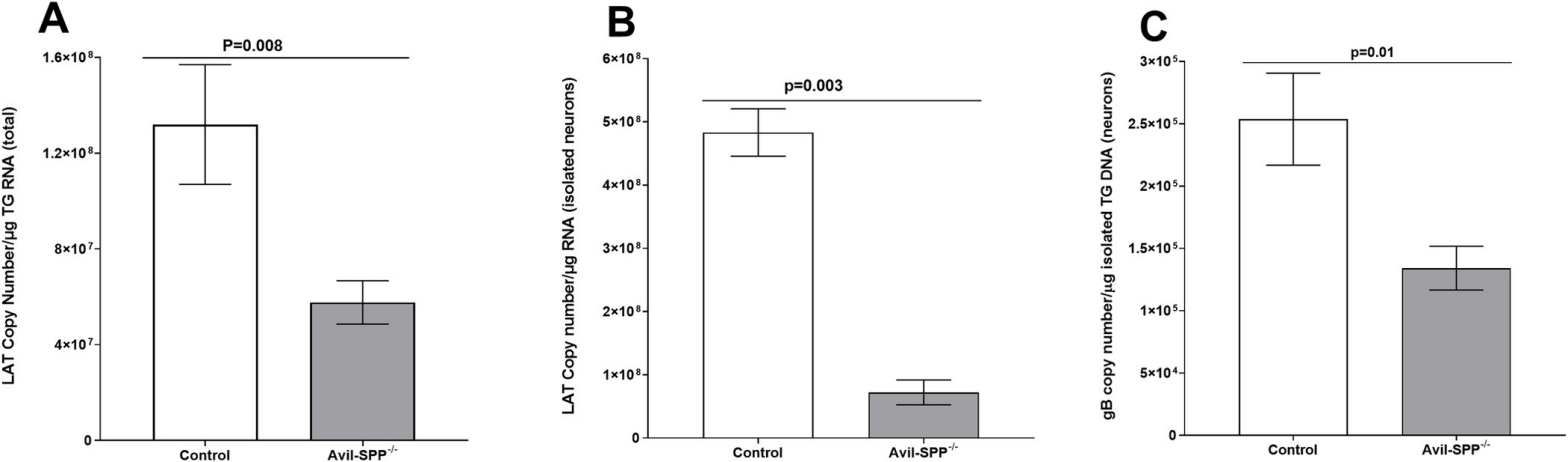
Effect of SPP deficiency on HSV-1 latency in TG of latently infected mice. Avil-SPP^-/-^ and control mice were ocularly infected with 2 X 10^5^ PFU/eye of HSV-1 strain McKrae. TG were harvested on day 28 PI. Neuronal fractions and total TG RNA or TG genomic DNA were isolated as described in Materials and Methods. LAT RNA or gB DNA copy numbers were measured by qRT-PCR and qPCR, respectively, using a standard curve generated with pAC-gB1 (for gB DNA) and pGEM-5317 (for LAT RNA). GAPDH expression was used to normalize relative levels of gB DNA and LAT RNA expression. Neuronal RNA was isolated from four combined TG, while total TG RNA was isolated from individual TG. Each bar represents the mean ± SEM from 12 mice for TG RNA and neuronal RNA and from 22 mice for total TG RNA.

During transition from lytic infection to latency in neurons of infected mice, rabbits, and humans, expression of more than 80 HSV-1 genes is drastically modified, and LAT is the only gene product consistently detected in abundance during latency [37,38,47–49]. To determine whether the absence of SPP in neurons of infected mice affects latency, Avil-SPP^-/-^ and control mice were infected ocularly as above and TG from infected mice were isolated on day 28 PI. Neurons were isolated for RNA and DNA extraction from some infected TG, while total RNA was isolated from other infected mouse TG. Isolated neuronal RNA and total TG RNA were used to quantify LAT RNA copy number, while DNA from infected neurons was used to quantify gB DNA copy number. Cellular GAPDH RNA and DNA were used as internal controls. The amount of LAT RNA during latency in total TG extract of Avil-SPP^-/-^ mice was significantly lower than in control mice (Fig 6A; p = 0.008, Fisher’s exact test). Similarly, the amount of LAT RNA in isolated neurons of infected Avil-SPP^-/-^ mice was significantly lower than in control mice (Fig 6B; p<0.0001, Fisher’s exact test). As expected, LAT copy number per μg of RNA from isolated neurons was higher than from total TG RNA (Fig 6, compare panels A and B). Finally, we compared the amount of gB DNA in isolated neurons of mice lacking SPP and control mice. Similar to the above results with lower levels of LAT in Avil-SPP^-/-^ mice, Avil-SPP^-/-^ mouse neurons had significantly less gB DNA than isolated neurons of control mice (Fig 6C; p = 0.014, Fisher’s exact test). These results suggest that in the absence of SPP in peripheral neurons, latency in TG of HSV-1 infected mice is reduced, which is consistent with our previous report in mice globally depleted of SPP using tamoxifen treatment [22].

### Absence of SPP in isolated neurons from Avil-SPP^-/-^ mice but not total TG significantly reduced CD8α and PD-1 expression during HSV-1 latency

**Fig 7:**
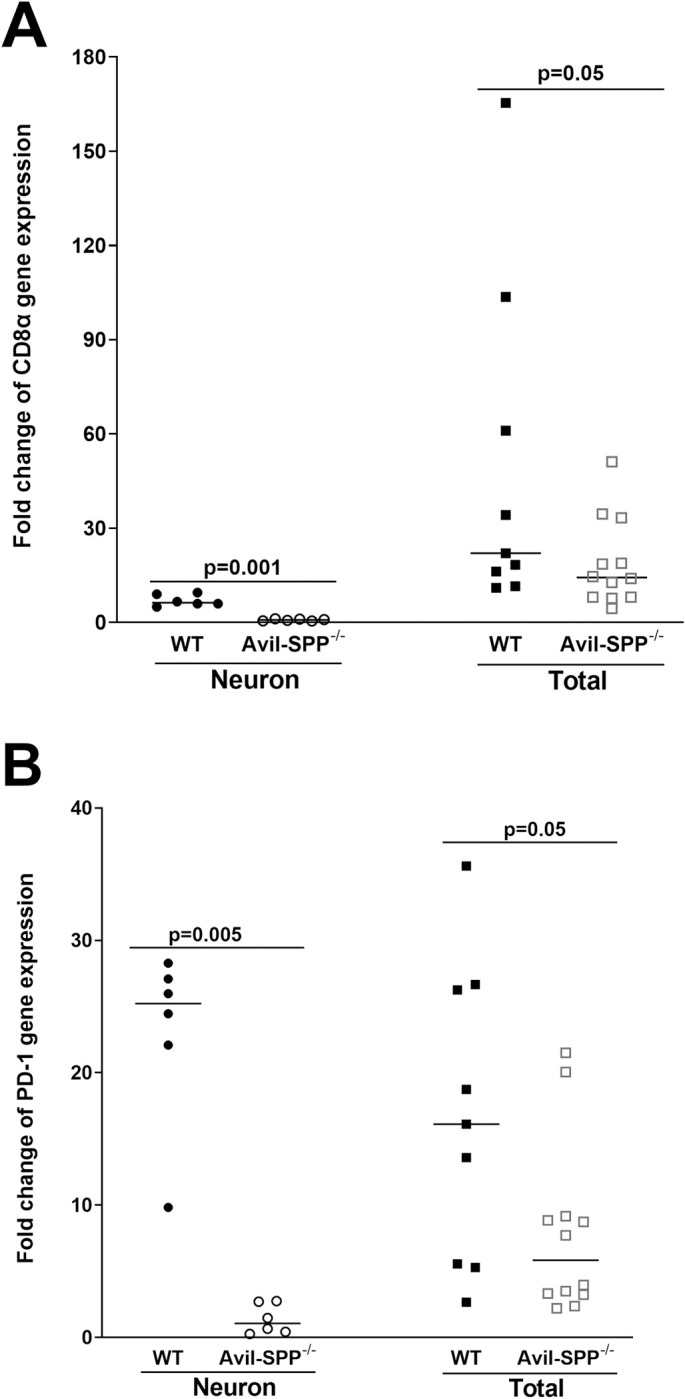
Effect of SPP deficiency on T cell exhaustion in TG of latently infected mice. Isolated RNA from total TG or neuron fractions (see Fig 5) were used to measure expression of CD8a (panel A) and PD-1 (panel B) transcripts by qRT-PCR. CD8a and PD-1 expression in TG of uninfected naive mice (control) was used as a baseline to estimate relative expression of each transcript in TG of infected mice. GAPDH expression was used to normalize relative expression of each transcript. Neuronal RNA was isolated from four combined TG, while total TG RNA was isolated from individual TG. Each bar represents mean ± SEM from 12 mice for TG and neuronal RNA and from 22 mice for whole TG.

The above results suggested that the absence of SPP affects LAT expression and gB DNA in TG of latently infected mice compared with control mice (Fig 6). Previously we had shown that lower latency in TG of latently infected mice correlated with lower levels of CD8α and PD-1 [50–52], thus, we looked whether lower latency in Avil-SPP^-/-^ mice affects expression levels of CD8α and PD-1 in TG of latently infected mice. RNA isolated from neurons and total TG of Avil-SPP^-/-^ and control mice (see Fig 6) were used to measure CD8α and PD-1 transcript levels by qRT-PCR. Results are presented as “fold” increase over baseline mRNA levels in TG of naive mice for each group. GAPDH mRNA in each sample was used as an internal control. Expression levels of CD8α transcript in isolated neurons of Avil-SPP^-/-^ mice was significantly lower than control mice (Fig 7A, p = 0.001, left panel, Fisher’s exact test), while the expression levels of CD8α transcript in total TG of Avil-SPP^-/-^ mice was similar to that of control mice (Fig 7A, p = 0.05, right panel, Fisher’s exact test). PD-1 transcript level also was significantly lower in isolated neurons from Avil-SPP^-/-^ mice compared with control mice (Fig 7B, p = 0.005, left panel, Fisher’s exact test). However, similar to CD8α levels in total TG, the level of PD-1 expression in total TG RNA from Avil-SPP^-/-^ mice was also similar to that of control mice (Fig 7B, p = 0.05, right panel, Fisher’s exact test). These results suggest that absence of neuronal SPP does affect expression levels of CD8α and PD-1 in isolated neurons during latent infection and this strongly correlates with lower level of latency as we reported previously [50–52].

### Absence of neuronal SPP protects from virus reactivation in an explant reactivation model

**Fig 8:**
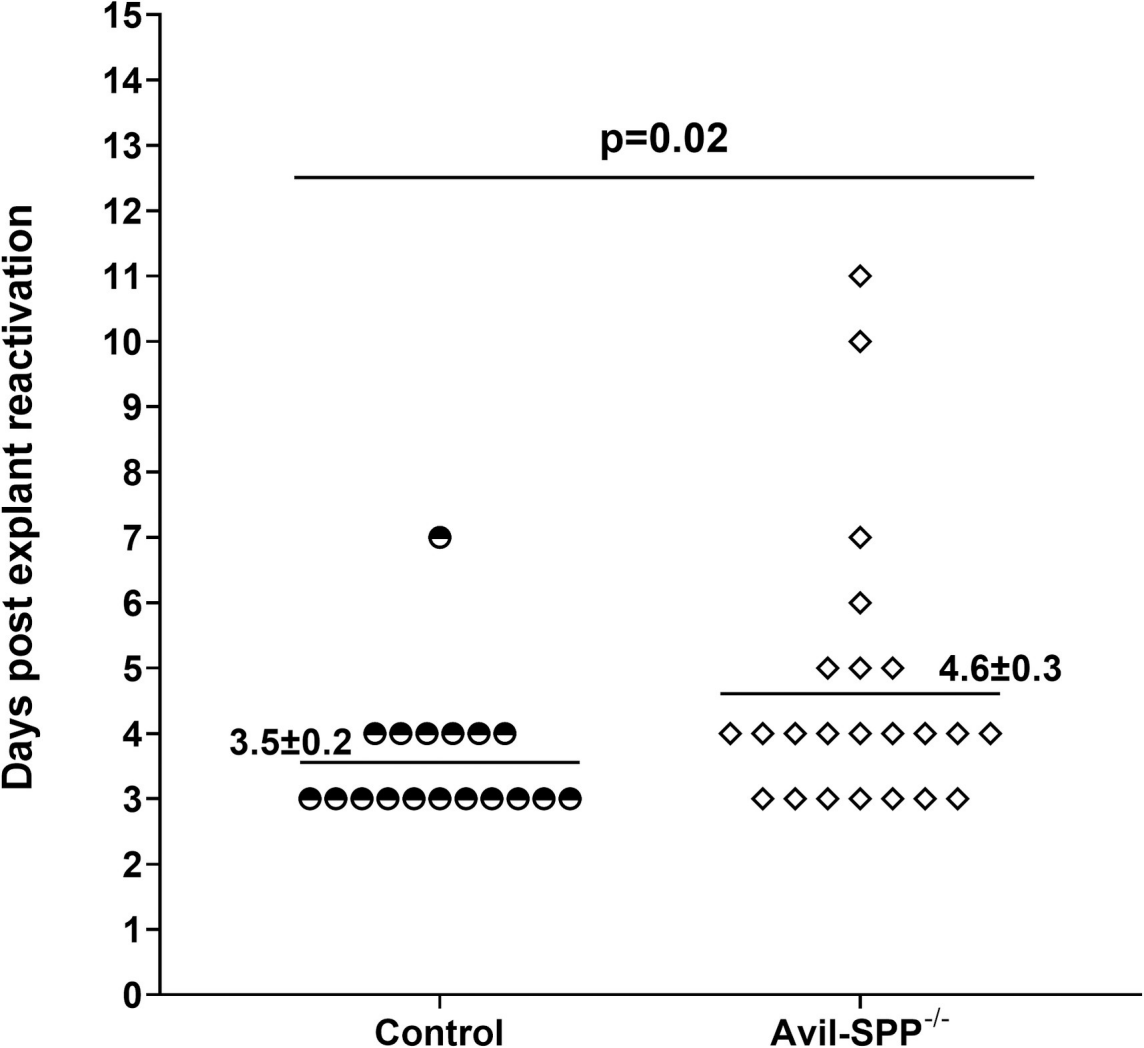
Effect of SPP deficiency on virus reactivation in infected mice. Avil-SPP^-/-^ and control mice were ocularly infected with 2 X 10^5^ PFU/eye of HSV-1 strain McKrae. TG from infected mice were harvested on day 28 PI and the explant reactivation assay was performed. Data points indicate the day of virus reactivation. Each point is the mean ± SEM from 34 TG.

The qRT-PCR analyses described in Fig 6 suggested that the absence of SPP in peripheral sensory neurons reduced LAT expression in the TG of latently infected mice. Thus, we asked whether reduced LAT levels correlated with reduced reactivation of latent virus. To test this, 17 Avil-SPP^-/-^ or control mice per group were ocularly infected with 2 X 10^5^ PFU/eye of HSV-1 strain McKrae. On day 28 PI, individual TG from infected mice were harvested and the kinetics of virus reactivation was measured in explanted TG. Average reactivation time for Avil-SPP^-/-^ mice was 4.6 ± 0.3 days, while control mice reactivated significantly faster, at an average of 3.5 ± 0.2 days (Fig 8, p = 0.02, Fisher’s t test). In addition, 32.4% of the TG from Avil-SPP^-/-^ mice did not reactivate at all in comparison to only 10% of TG from control mice. These differences were highly significant between the groups (p<0.01, Fisher’s exact test). Together, these results suggest that absence of SPP in neurons drastically increases time to reactivation and decreases the number of reactivation events, and thus may protect infected mice from reactivation. These results also confirm our previous studies showing that lower virus load in the TG of latently infected mice strongly correlates with slower reactivation in TG of latently infected mice [50–52].

## Discussion

After primary HSV-1 infection the virus establishes latency in trigeminal ganglia of the infected host with subsequent episodes of viral reactivation to the primary site of infection, which is the main cause of HSV-1-induced eye disease [53,54]. HSV-1-induced corneal scarring (CS), broadly referred to as herpes stromal keratitis, is the leading cause of infectious blindness in developed countries and in the U.S., with approximately 30,000 people suffering recurrent ocular HSV episodes annually, requiring doctor visits, medication, and in severe cases, corneal transplants [55–60]. After an initial episode, recurrence rates of ocular HSV increase over years of infection [61] with the immune response to the virus contributing to eye disease [62–64].

Our previous studies have established a critical role for SPP binding to gK during HSV-1 ocular infections. HSV-1 gK contributes to severe CS and facial dermatitis following ocular HSV-1 infection that occurs independent of virus or mouse strain [3,9,10,65]. We have also shown that gK binds to SPP and that SPP is required for HSV-1 infectivity *in vitro* [20,21] and *in vivo* [22]. Thus, the interaction of gK with SPP contributes to its pathogenic functions *in vivo*.

Since SPP is an essential gene, we previously used a tamoxifen inducible Cre recombinase driven by the ubiquitously expressed ROSA26 promoter to globally delete SPP in mice [22]. We found decreased virus replication and reduced latency and reactivation in mice lacking SPP. However, while tamoxifen treatment had no adverse effects, mice globally depleted of SPP showed early stunted growth [22]. Additionally, peripheral nerves play a cardinal role in virus transport to the CNS of the infected host, and eventually leading to establishment of latency and subsequent reactivation. Therefore in this study, we specifically deleted SPP in peripheral sensory neurons using the Advillin-Cre system.

Advillin is an actin binding protein with exclusive expression in peripheral sensory neurons during development and adulthood. It is expressed at high levels in dorsal root ganglia (DRG), TG, vestibulocochlear ganglia, glossopharyngeal ganglia, and vagus ganglia, but at low levels in the brain [41,66–69]. Thus, Advillin provides an excellent model to delete SPP in peripheral sensory neurons without affecting expression in non-neuronal cells, and the Avil-SPP^-/-^ mice generated in this study allowed us to directly evaluate the role of SPP during HSV-1 infection without involving other cell types. In contrast to the embryonic lethality of mice lacking SPP globally [22,34], or the stunted growth seen in conditional SPP knock out mice, deletion of SPP in peripheral sensory neurons of Advillin-Cre mice had no obvious side effects. As expected, SPP levels were significantly reduced in TG and isolated neurons of Avil-SPP^-/-^ mice compared with control mice before or after infection.

We previously established a critical role for gK binding to SPP during HSV-1 ocular infections [21,22]. Our current study focused on the absence of SPP in peripheral sensory neurons to understand the importance of SPP in these cells, especially related to virus replication in the TG of infected mice. We found that the absence of SPP did not affect virus replication in the eyes of infected Avil-SPP^-/-^ mice compared with control mice. This is in contrast with our *in vitro* studies using SPP dominant-negative constructs and SPP shRNA [20] and with our *in vivo* study of mice globally depleted of SPP after tamoxifen treatment [22], where we observed significantly less virus replication upon SPP depletion. This discrepancy is likely due to the presence of SPP in all non-neuronal cells of the eyes in Avil-SPP^-/-^ mice, while tamoxifen treatment depleted SPP in all cell types in the eye. We previously showed that global depletion of SPP was associated with significantly lower levels of gB, gK, and ICP0 transcripts in total TG isolated from mice on day 5 PI [22]. While levels of these transcripts in total TG RNA extracts did not differ in our current study, significantly lower levels of gB, gK, and ICP0 transcripts were detected in neurons isolated from TG of infected Avil-SPP^-/-^ mice than in control mice on both days 2 and 5 PI. This result is consistent with the lower levels of these transcripts seen in tamoxifen treated mice [22], suggesting the importance of SPP in HSV-1 infectivity. Similar to global depletion of SPP by tamoxifen treatment [22], the absence of SPP in peripheral sensory neurons of infected mice did not affect their susceptibility to ocular infection with the virulent HSV-1 strain McKrae. Furthermore, and as we reported previously [22], after infection of mice lacking SPP in their peripheral sensory neurons, the level of eye disease was similar to that in control mice.

**Fig 9:**
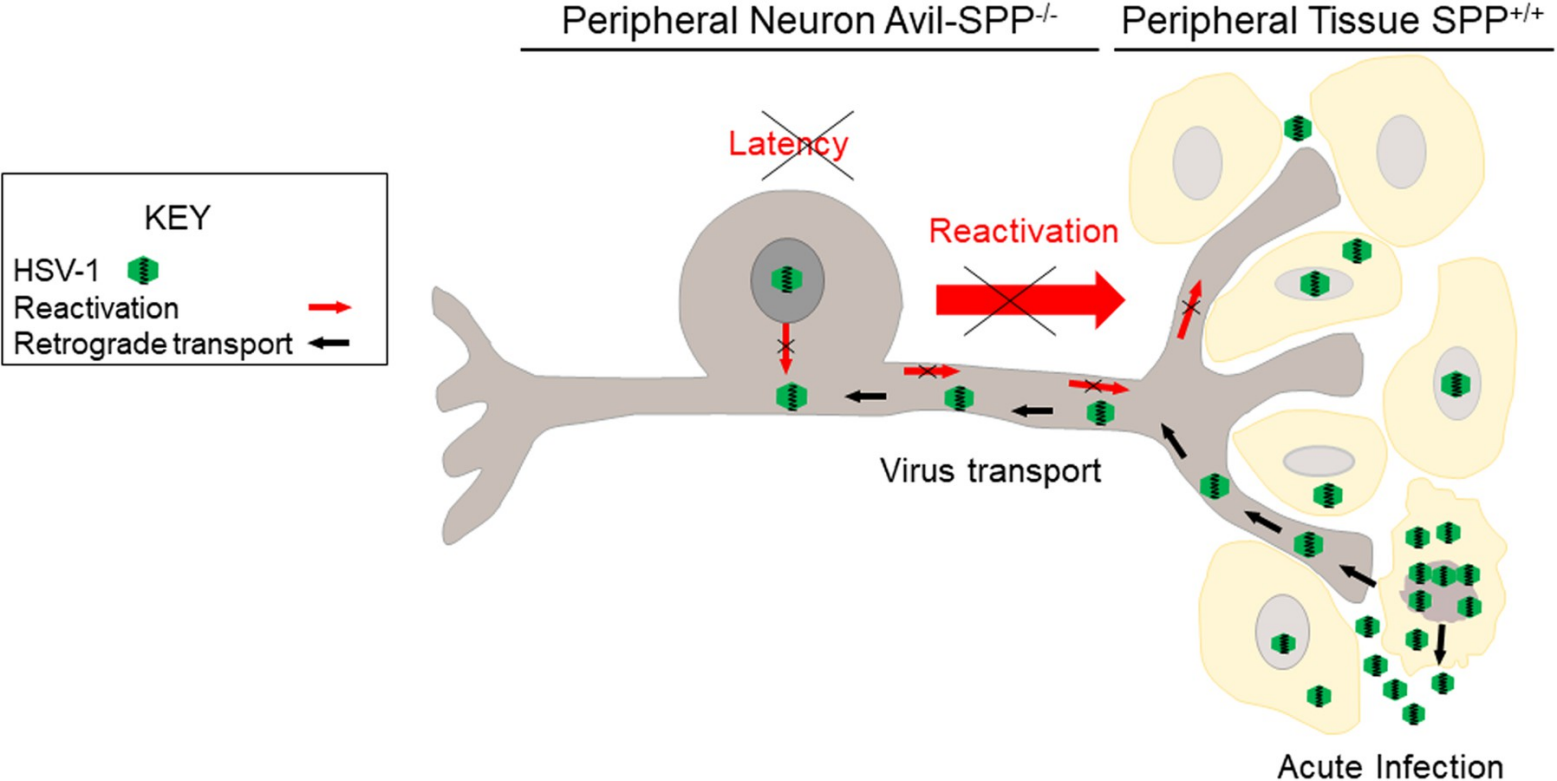
Schematic diagram of the absence of SPP in peripheral sensory neurons of Avil-SPP^-/-^ mice and its effect on latency-reactivation, not primary ocular infection. SPP is present in the eye of Avil-SPP^-/-^ mice and virus replication is not affected. As shown, HSV-1 replicates normally in peripheral tissue due to the presence of SPP and moves to peripheral neurons via retrograde transport. However, knockdown of SPP results in inhibition of HSV-1 replication in Avil-SPP^-/-^ neurons and blocking/reduction of latency establishment, thus affecting transport of virus to site of infection and reactivation.

During latency HSV-1 gene expression is curtailed with LAT being the only gene product that is expressed abundantly in neurons of infected mice, rabbits, and humans [37,38,47–49]. In this study we showed significantly less LAT expression and gB DNA in neurons isolated from Avil-SPP^-/-^ mice than in control mice. LAT expression was also lower in TG of Avil-SPP^-/-^ mice than in control mice. Similar results were seen when we depleted SPP using tamoxifen in our previous published study [22]. Consistent with lower levels of LAT and gB in isolated neurons and TG of mice lacking SPP than in control mice, we also found lower levels of CD8α and PD-1 expression in isolated neurons but not in whole TG of mice lacking SPP. We previously showed that lower levels of latency strongly correlates with lower levels of CD8α and PD-1 expression and thus, T cell exhaustion [50]. In addition to increasing levels of latency and T cell exhaustion, the HSV-1 LAT gene is known to play a critical role in enhancing the reactivation phenotype in TG of the infected host [47,70–72]. LAT deletion mutant viruses have a reduced reactivation phenotype in both mice and rabbits [47,70–72]. In this study, the absence of SPP expression in TG of Avil-SPP^-/-^ mice delayed time of reactivation and prevented reactivation in infected Avil-SPP^-/-^ mice compared with control mice. Thus, reduced expression of viral transcripts in Avil-SPP^-/-^ mouse neurons during primary infection correlates with lower levels of latency-reactivation than seen in control mice. Schematic model of how gK binding to SSP mediates establishment of latency in TG neurons is shown in Fig 9.

In summary, we have provided the first evidence that the absence of SPP specifically in peripheral sensory neurons reduces virus replication and latency-reactivation in TG of infected Avil-SPP^-/-^ mice as compared with control mice. Moreover, lack of SPP in peripheral neurons was not associated with any adverse effects on the host. Hence, we conclude that latency-reactivation levels are regulated by the interaction of gK with SPP. Consequently, blocking gK-SPP interaction may reduce the level of latency and subsequent reactivation, which is the primary cause of HSV-1-induced pathology.

## Material and methods

### Ethics statement

All animal procedures were performed in strict accordance with the Association for Research in Vision and Ophthalmology Statement for the Use of Animals in Ophthalmic and Vision Research (https://www.arvo.org/About/policies/statement-for-the-use-of-animals-in-ophthalmic-and-vision-research/) and the NIH *Guide for the Care and Use of Laboratory Animals*. The animal research protocol was approved by the Institutional Animal Care and Use Committee of Cedars-Sinai Medical Center (protocol no. 9129).

### Cells, viruses, and mice

The RS (rabbit skin) cell line was cultured in minimal essential medium plus 5% fetal bovine serum and maintained as described previously [10]. Triple-plaque-purified HSV-1 strain McKrae was grown in RS cell monolayers as described previously [73]. HSV-1 strain McKrae is a virulent strain of virus that infects mice efficiently without corneal scarification. SPP^flox/flox^ mice were generated and maintained in-house as previously described [22]. Briefly, the SPP conditional knockout Avil-SPP^-/-^ strain was generated by crossing SPP^flox/flox^ mice with Advillin-Cre mice (#32536; Jackson Laboratory). Since female Advillin-Cre mice have weak Cre expression in oocytes, which may result in germline knockout [74], only male Avil-SPP^-/-^ mice were used to breed with female SPP^flox/flox^ mice to generate pups. In each breeding cycle, the genotype of Avil-SPP^-/-^ mice was confirmed by PCR and only confirmed positive mice (both male and female) were used for experiments. Although gender has been shown to influence survival of BALB/c and 129/Sv//Ev mice after HSV-1 ocular infection [75,76], we did not detect any differences in survival between male and female Avil-SPP^-/-^ or wt mice. These differences between our study and previous studies are probably due to susceptibility of both BALB/c and 129/Sv//Ev mice to HSV-1 infection compared to the C57BL/6 background mice which are refractory to HSV-1 infection. Previous studies have shown no differences between male and female mice in terms of virus replication in the brain [75], virus clearance from the TG or reactivation [76], in viral titers in tear films or affected tissues, in immune cell infiltration, or in clinical symptoms [77]. Similar to these studies and in line with our previous and current studies, we did not detect any differences between infected male and female mice with regards to virus replication in the eye, eye disease, survival, latency or reactivation in mice with C57BL/6 background [78–87]. WT C57BL/6 mice were used as controls and were purchased from The Jackson Laboratory (Bar Harbor, ME) and bred in-house at Cedars-Sinai Medical Center.

### Primers for Avil-SPP^-/-^ mice genotyping

The following primers were used to screen Avil-SPP^-/-^ mice: SPP forward primer, TGCCTCCCGTTTAAGAGACC, and SPP reverse primer, GACTCATTCTCCCCGCTCTG. The wild-type allele product length is 605 bp, and the floxed allele product length is 688 bp according to The Jackson Laboratory protocol. The following primers were used to screen Advillin-Cre mice: GCGATCCCTGAACATGTCCATC (forward) and CCCTGTTCACTGTGAGTAGG (reverse), producing a PCR product length of 180 bp. The following primers were used to detect the SPP deletion: CGCTTCGGAGCATTGTGA (forward) and CCATGAGGAGCCAAGCC (reverse), producing a PCR product length of 270 bp.

### Ocular infection

Control and Avil-SPP^-/-^ mice (7 to 8 weeks old of both sexes) were infected ocularly in both eyes with 2 X 10^5^ PFU/eye of HSV-1 strain McKrae in tissue culture medium as an eye drop without corneal scarification as we described previously [10].

### Total TG RNA extraction

After ocular infection with 2 X 10^5^ PFU/eye of HSV-1 strain McKrae, TG from infected mice were collected on day 2, 5 or 28 PI. Isolated tissues were immersed in TRIzol reagent and stored at −80°C until processing. Corneas or TG from each animal were processed for RNA extraction as we described previously [12]. Isolated total RNA was reverse transcribed with random hexamer primers, and murine leukemia virus reverse transcriptase was provided in a High Capacity cDNA reverse transcription kit (Applied Biosystems, Foster City, CA) according to the manufacturer’s recommendations.

### Isolation of neurons from TG

To isolate neuron cells from TG, four TG were harvested and pooled from two mice in each of the four groups: Avil-SPP^-/-^ or control mice with and without infection. Pooled TG were then incubated in 1xHBSS with 20 mg/ml collagenase D and DNase 1 at 37°C for 1 hr to create a single cell suspension. Neuron cells were then isolated by negative selection using a neuron isolation kit (Miltenyi Biotec, # 130-115-389) following the company’s instructions. Briefly, cells were stained with the non-neuronal cell Biotin-Antibody cocktail (containing antibodies that bind to non-neuronal cells like astrocytes, oligodendrocytes, microglia, endothelial cells, fibroblasts, except erythrocytes) at 4°C for 5 min and then washed with 1xPBS, 0.5%BSA. Cells were spun down, resuspended, and incubated with anti-biotin microbeads for 10 min at 4°C. Neuron cells were separated by passing through an LS column under a magnetic field. Flow cytometric analysis was done to determine the percentage of positive neurons from isolated TG using Alexafluor 488-anti-Tubb3 antibody (Biolegend, #801203) or anti-NeuN antibody (Biolegend, #834502) followed by AlexaFluor 488 donkey anti mouse secondary antibody. Stained cells were gated as shown in S1 Fig. Our FACS analysis of isolated neurons showed purity greater than 92.9% and 94.7% following staining with anti-NeuN and anti-Tubb3 antibodies, respectively. This is similar to what was previously reported by the manufacturer (https://atcg.com.hk/wp-content/uploads/2017/04/a-novel-method-for-rapid-isolation-of-untouched-neurons-miltenyi-biotec.pdf). RNA or DNA was extracted from neurons as previously reported [12].

### TaqMan qRT-PCR

Sequences of the gB, gK, and ICP0 custom-made TaqMan primer sets used in this study were as follows. For gB, 5′-AACGCGACGCACATCAAG-3′ (forward), 5′-CTGGTACGCGATCAGAAAGC-3′ (reverse), probe 5’-FAM-CAGCCGCAGTACTACC-3′ (where FAM is 6-carboxyfluorescein). For gK, 5’-GGCCACCTACCTCTTGAACTAC-3’ (forward), 5′-CAGGCGGGTAATTTTCGTGTAG-3′ (reverse), probe 5′-FAM-CAGGCCGCATCGTATC-3. For ICP0, 5′CGGACACGGAACTGTTCGA-3′ (forward), 5′-CGCCCCCGCAACTG-3′ (reverse), probe 5′-FAM-CCCCATCCACGCCCTG-3′. GAPDH primers from Applied Biosystems (assay identifier, m999999.15_G1) were used as an internal control. Other TaqMan primers from Applied Biosystems used in this study were CD8a (Mm01182107_g1), SPP (Mm00468786_m1) and PD-1 (Mm00435532_m1).

Quantitative PCR (qPCR) was performed using a TaqMan gene expression assay kit in 384-well plates on an ABI QuantStudio 5 system (Applied Biosystems, Foster City, CA). Copy numbers for gB, gK, and ICP0 were calculated using standard curves generated using pAc-gB1 (for gB), pGem-gK1040 (for gK), pcDNA-ICP0 (for ICP0) and pGEM5317 (for LAT). Briefly, each plasmid DNA template was serially diluted 10-fold so that 1 μl contained from 10^3^ to 10^8^ copies of the desired gene that was then subjected to TaqMan PCR with the same set of primers as the test samples. Copy number of each reaction product was determined by comparing the normalized threshold cycle (*C*_*T*_) of each sample to the threshold cycle of the standard curve. The 2^−ΔΔ*CT*^ method was used to calculate fold change in gene expression compared to expression in uninfected controls.

### DNA extraction and PCR analysis for HSV-1 latency

Avil-SPP^-/-^ and control mice were infected as described above. DNA was isolated from homogenized individual TG on day 28 PI using the Dneasy Blood &Tissue Kit (Qiagen, Stanford, CA Cat. No. 69506) according to the manufacturer’s instructions. PCR analyses used the gB specific primers, 5’-AACGCGACGCACATCAAG-3’ (forward), 5’-CTGGTACGCGATCAGAAAGC-3’ (reverse), and probe 5’-FAM-CAGCCGCAGTACTACC-3’. Amplicon length for this primer set is 72 bp. Relative gB DNA copy numbers were calculated using standard curves generated from plasmid pAc-gB1. GAPDH was used to normalize transcripts in all experiments.

### IHC staining and image analysis

TG were harvested from control or Avil-SPP^-/-^ mice before or after infection, frozen in OCT compound and stored at -80°C until processing. Frozen tissue was sectioned using a MicroM HM 550 Cryostat. Tissue sections were fixed with 4% paraformaldehyde, washed with 1x phosphate-buffered saline (PBS), and then permeabilized with 0.3% Triton X-100 in PBS. Slides were blocked using 1x sea blocker (Thermo Scientific, Rockford, IL) plus 5% horse serum for 1 hr at 25°C, then incubated with anti-SPP antibody (Bethyl Laboratory, A304-404A) or anti-HSV-1 antibody (GeneTex, GTX26506) in blocking buffer at 4°C overnight. Slides were washed 3X with 1xPBS and incubated with biotin-conjugated secondary antibody for 2 hr at 25°C. Slides were washed 4X with 1xPBS and developed using a Vector VIP substrate kit according to company’s instructions (Vector Laboratories, Burlingame, CA. Cat# SK-4600). Slides were mounted with Permount mounting medium (Fisher Scientific, SP15-100) and image acquisition and data analysis were done using a Leica DM4000 microscope (Leica Microsystems, Buffalo Grove, IL). Density of the staining was calculated by dividing mean gray value by area of each image measured by Image J software [88].

### Titration of HSV-1 in tears

Tear films were collected from both eyes of infected mice from days 1 to 5 PI, using a cotton applicator. Each swab was placed in 1 ml of tissue culture medium and stored at –80°C until processing. The amount of virus in the medium was determined by standard plaque assay using RS cells as we described previously [12].

### HSV-1 induced eye disease

The severity of corneal scarring was assessed by examination under a slit-lamp biomicroscope on day 28 PI. The examination was conducted by investigators blinded to the treatment regimen of the mice and scored according to a standard 0 to 4 scale (0 = no disease, 1 = 25%, 2 = 50%, 3 = 75%, and 4 = 100% involvement) as we described previously [9].

### In vitro explant reactivation assay

TG from infected mice were harvested 28 days PI, cultured in 1.5 ml tissue culture medium, and the reactivation assay was performed as we described previously [12]. Briefly, a 100-μl aliquot was collected from each culture daily and used to infect RS cell monolayers, which were monitored daily for 5 days for the appearance of cytopathic effect (CPE) to determine the time that reactivated virus first appeared from each TG. Because media from the explanted TG cultures were plated daily, the time at which reactivated virus first appeared in explanted TG cultures could be determined.

### Statistical analysis

Fisher’s exact test and Mann Whitney test were performed using the computer program Instat (GraphPad, San Diego, CA). Results were considered statistically significant at a *P* value of <0.05.

## Supporting information

S1 Fig Analysis of neuron population in isolated TG by FACS. (TIF)
