# Supplementary figures and images for: Absence of signal peptide peptidase in peripheral sensory neurons affects latency-reactivation in HSV-1 ocularly infected mice
Source: PLoS Pathog. 2022 Jan 31;18(1):e1010281. doi: 10.1371/journal.ppat.1010281 (PMC8830783; doi:10.1371/journal.ppat.1010281)

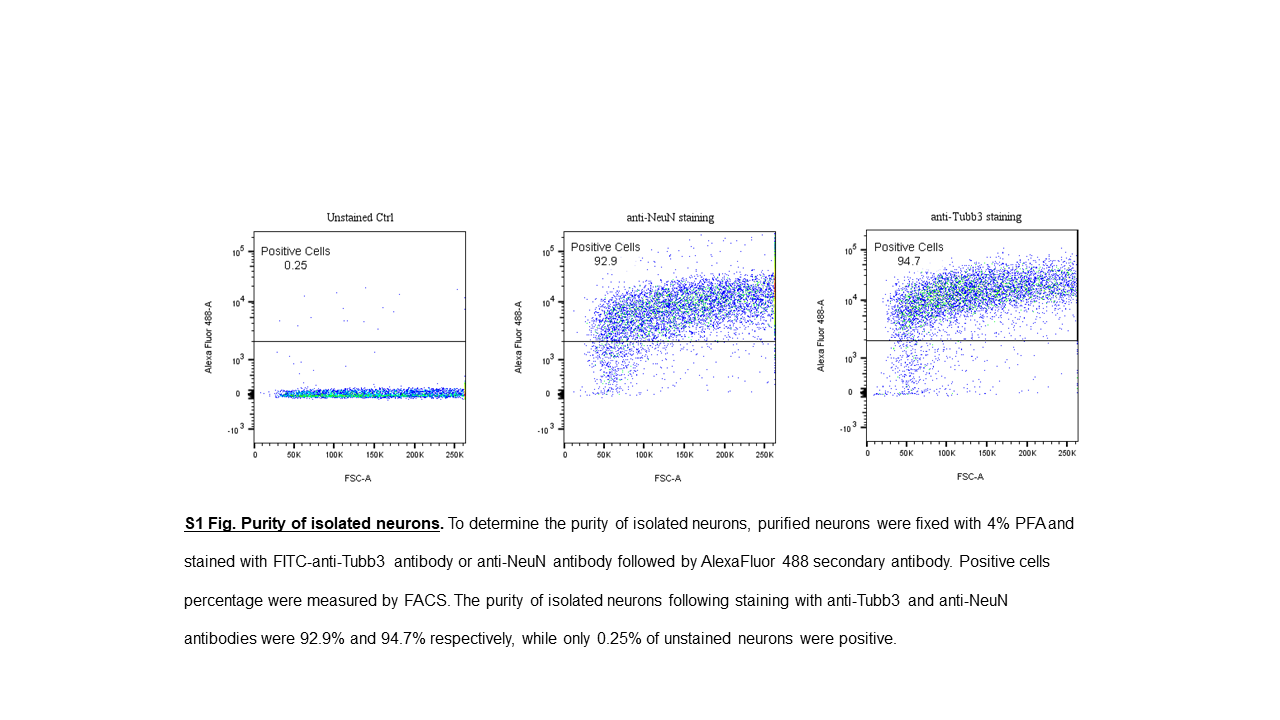

Supplement: S1 Fig — (TIF) [file ppat.1010281.s001.tif]
